# A highly integrated DNA nanomachine operating in living cells powered by an endogenous stimulus†
†Electronic supplementary information (ESI) available: Experimental details, Fig. S1–S13, and Tables S1 and S2. See DOI: 10.1039/c8sc00049b


**DOI:** 10.1039/c8sc00049b

**Published:** 2018-02-23

**Authors:** Pei-Qiang Ma, Cheng-Pin Liang, He-Hua Zhang, Bin-Cheng Yin, Bang-Ce Ye

**Affiliations:** a Collaborative Innovation Center of Yangtze River Delta Region Green Pharmaceuticals , College of Pharmaceutical Sciences , Zhejiang University of Technology , Hangzhou 310014 , Zhejiang , China; b Lab of Biosystem and Microanalysis , State Key Laboratory of Bioreactor Engineering , East China University of Science & Technology , Shanghai , 200237 , China . Email: binchengyin@ecust.edu.cn

## Abstract

Synthetic molecular machines have received increasing attention because of their great ability to mimic natural biological motors and create novel modes of motion. However, very few examples have been implemented with real autonomous movement inside living cells, due to the challenges of the driving force and highly integrated system design. In this work, we report an elegant, highly integrated DNA nanomachine that can be powered by endogenous ATP molecules and autonomously operated inside living cells without any auxiliary additives. It assembles all components on a single gold nanoparticle (AuNP) including a hairpin-locked swing arm encoding a start triggered by an intracellular target molecule and a two-stranded DNA track responding to the motion of the swing arm. When the intracellular target activates the nanomachine *via* the unlocking swing arm, the machine autonomously and progressively operates on the established DNA track *via* intramolecular toehold-mediated strand migration and internal ATP binding. This paper also demonstrates the machine's bioanalytical application for specific microRNA (miRNA) imaging in living cells.

## Abstract 2

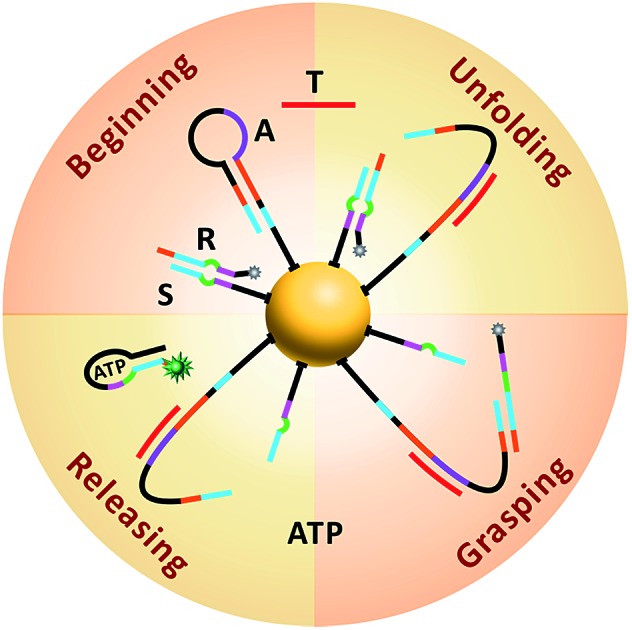
An elegant, highly integrated DNA nanomachine powered by endogenous ATP molecules was developed for specific microRNA imaging in living cells.

## Introduction

A vast range of natural protein motors exist in the biological world, and they participate in significant biological processes, such as myosin in muscle contraction,^1^ kinesin and dynein in cellular cargo transport^2^,^3^ and DNA polymerase in DNA synthesis.^4^ Inspired by these biological motors, researchers have attempted to fabricate various artificial molecular machines and motors that can be precisely controlled and exploited on the nanometer scale, simultaneously connecting molecular systems to the macroscopic world. Among them, synthetic DNA-based machines, with a simple DNA structure, high programmability and predictability of Watson–Crick base pairing, good structural robustness and low cost of synthesis, exhibit significant advantages that enable the fully *de novo* design of diverse and powerful devices. So far, many DNA machines, such as walkers,^5^–^12^ rolling motors,^13^ tweezers,^14^ gears,^15^ springs,^16^ robots^17^–^20^ and transporters^21^ have been created to mimic the movement of protein motors or to have distinct behaviour patterns, and successfully tested in cell-free settings. Fabricating DNA nanodevices that could practically operate inside living cells to execute designated tasks, such as *in vivo* imaging, biological computing and autonomous theranostic application, has long been a challenging goal. It is noteworthy that most of the reported sophisticated DNA machines are designed with complicated DNA-origami tracks, large-scale nanomaterials or complex motion patterns, which have usually limited their potential applications in living cells.

Another great challenge for cellular DNA nanomachines is the design of a suitable driving force to fuel the autonomous operation in a simple and effective manner. Unlike ATP hydrolysis used in protein motors as an energy source, other means, such as protein enzymes,^10^–^12^ DNA strands,^22^,^23^ pH variation,^24^–^26^ metal ions^27^ and light^28^–^30^ have been explored to power DNA nanomachines, with the result that most of the reported nanomachines need exogenous additives to drive their motion. For example, our group developed an entropy-driven DNA nanomachine by transferring a DNA fuel strand *via* liposome into living cells to power the machine.^22^ Le *et al.* developed a DNAzyme motor fueled by DNAzyme catalyzed substrate cleavage with the addition of Mn^2+^ to cells.^27^ These nanomachines have achieved significant advances towards biological applications *in vivo*, but the external co-delivery of fuels, addition of related supplementary components or variations in environmental factors complicate the design and handling procedures. In addition, the asynchronous delivery of components into cells may decrease the control of the spatiotemporal distribution of core components of DNA nanomachines and the auxiliary components in living cells. Although pH-driven nanomachines can spontaneously respond to environmental pH variation, their biological application might be limited due to their intrinsic switch mode. Thus, it is desirable to develop more integrated DNA nanomachines with an endogenous driving force to execute diverse biological tasks *in vivo*.

Herein, we have progressed beyond our previous design^22^ to develop an ATP-fueled DNA nanomachine with a simpler yet elegant design. Notably, ATP is the main source of energy for most cellular processes, and is ubiquitous in the cytoplasm and nucleoplasm of every cell. Thus, we employ endogenous ATP as a fuel by coupling an ATP aptamer to drive the autonomous motion of the machine for executing the task of sensing target miRNA *in vivo*. The proposed machine is highly integrated with only three components, a hairpin-locked swing arm and a two-stranded DNA track assembled on a single gold nanoparticle (AuNP). A start switch, using a specific endogenous miRNA as an initiator, is embedded in the hairpin-locked swing arm. The DNA track is integrated with an ATP aptamer for response to endogenous ATP. After the intracellular uptake of the DNA nanomachine, the interaction of the miRNA target and hairpin-locked swing arm can specifically initiate an autonomous motion of the swing arm along the three-dimensional DNA–AuNP track inside living cells with a constant supply of free energy from the binding of ATP and its aptamer (track component), thus providing amplified signal production for sensing target miRNA with low abundance. It should be noted that all nanomachine components assembled together provide enhanced local concentrations due to their close proximity to the DNA pairing reaction and the corresponding rapid reaction rate and efficiency.^11^

## Results and discussion

### Principles of the ATP-powered DNA nanomachine

**Fig. 1:**
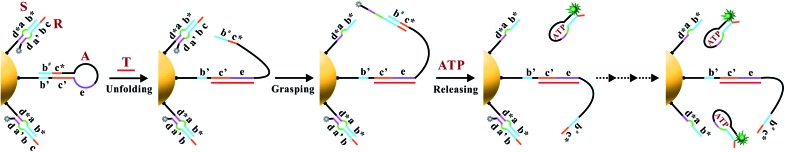
Schematic illustration of the motion of the proposed ATP-powered DNA nanomachine. The dotted arrows represent the repeated processes of grasping and releasing.

The working principle of the proposed nanomachine is illustrated in Fig. 1. It contains an AuNP assembled with a high density of two-stranded substrate complexes (S/R) as tracks and a hairpin-locked swing arm (A). The functional sequence of R is designed to include a toehold (domain **c**) and 27-nt ATP aptamer (divided into domains **b**, **a′**, and **d**, and a 5-nt tail) with carboxyfluorescein (FAM) at the 3′ end for the signal output. The sequence S was modified with a thiol at the 5′ end for direct immobilization onto the AuNP surface. A has an elaborately designed stem-loop hairpin structure, containing a complementary segment (domains **c′** and **e**, located in the stem and loop, respectively) to a specific target (T), and trapped domains **b^#^** and c* in the stem. In addition, A is modified with a thiol at the 3′ end for tethering to the AuNP. The proposed DNA nanomachine is designed in the initial state as follows. Hundreds of S/R complexes are tethered to AuNPs with FAM fluorophores of R quenched by AuNPs. R is firmly bound to S even in the presence of ATP because most of its ATP aptamer sequence is occupied by S. Meanwhile, dozens of A maintain their stable hairpin structure by locking domains **b^#^** and c* on their stem. After the cellular uptake of the nanomachine, the intracellular target T specifically unfolds the hairpin structure of A and turns its end into a free swing arm with exposed domains **b^#^** and c*. Subsequently, A performs a domain c*-mediated branch migration reaction that grasps R from S and exposes more ATP aptamer sequence of R. Then, free ATP molecules in living cells spontaneously bind to R *via* the ATP aptamer, resulting in the release of an ATP/R complex with concurrent fluorescence restoration of FAM in R and simultaneous release of the free-swinging A to spread along the DNA–AuNP track. Accordingly, the activated A automatically grasps new R from the S/R track and triggers a continuous fluorescence signal output due to the formation of the ATP/R complex. Thus, by measuring the FAM fluorescence intensity in living cells, the proposed DNA nanomachine presents a simple and efficient mechanism for the specific detection of intracellular biomolecules of interest.

### Optimization of the two-stranded DNA track (S/R)

**Fig. 2:**
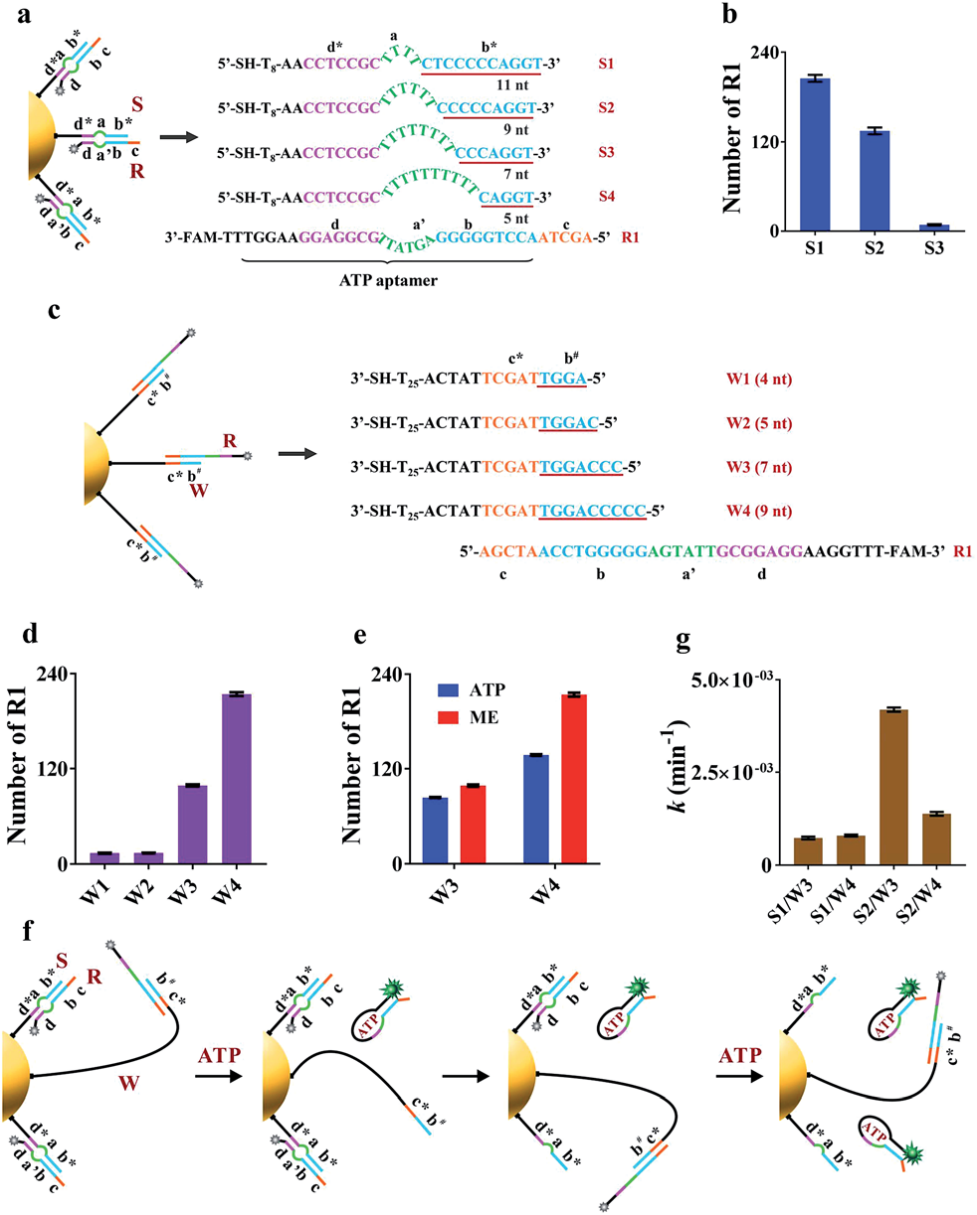
Investigation of the best combination of domain **a** on S and domain **b^#^** on A. Domain **a** on S and domain **b^#^** on A are two critical factors related to grasping and releasing motions. (a) Different S with R1 were assembled on AuNPs. Detailed sequence information of S1–S4 with different lengths of domain **a**. (b) The number of assembled R1 on every single AuNP with different S. (c) Different linear strands (W) with R1 were assembled on AuNPs. Detailed sequence information of W1–W4 with different lengths of domain **b^#^**. (d) The number of assembled R1 on every single AuNP with different W. (e) The number of released R1 on every single AuNP by ATP and ME. 0.1 nM R1/W-AuNPs were incubated with 10 mM ATP or 20 mM ME for 1 h or 12 h, respectively. (f) Schematic illustration of the working mechanism of the nanomachine assembled with W. (g) Rate constants *k* of four nanomachines (S1/R1/W3-AuNP, S1/R1/W4-AuNP, S2/R1/W3-AuNP and S2/R1/W4-AuNP). Error bars represent the standard deviation from three independent experiments.

The key to the motion of our nanomachine is effective grasping of R from the S/R complex by free domains **b^#^** and c* of A and subsequent R release by ATP. To realize this, we first rationally optimized R and S. The sequence information for oligonucleotides used in this work is shown in Table S1.† Domain **c** of R was designed as AGCTA based on our previous work,^22^ because the 5-nt toehold length of the AuNP-based DNA track was capable of activating toehold-mediated strand displacement. S is expected to tightly bind to R *via* pairing with the ATP aptamer to prevent ATP binding, and effectively release R when free A grasps R *via* domain c*-mediated strand displacement. To balance these two processes, we introduced an internal-loop region between the complementary region in S/R using polythymine (polyT) as domain **a** and the 7 base-pair linkage of domains d* and **d**, which is not strong enough to hold the linkage when the binding between domains b* and **b** is destroyed. As shown in Fig. 2a when the length of polyT loop in S1 to S4 increases from 4 to 10 nt, the number of base pairs between domains b* and **b** decreases from 11 to 5, correspondingly. The melting temperatures (*T*_m_) of the hybrids of S1/R1, S2/R1, S3/R1, and S4/R1 are predicted to be 58.8 °C, 56.1 °C, 46.8 °C, and 37.3 °C, respectively. S4 was first ruled out due to the intracellular temperature of *ca.* 37 °C. Then we assembled S1/R1, S2/R1 and S3/R1 on AuNPs, respectively, and calculated the number of R1 tethered by different S on every single AuNP based on mercaptoethanol (ME)-induced Au–S bond dissociation (see procedure in the ESI†). Fig. 2b shows that S3 had the least number of R1 strands (*ca.* 9 strands per AuNP) because the S3/R1 complex with additional 7 base pairs (bp) between domains b* and **b** is not stable enough to maintain the double-stranded structure. On the contrary, S1 and S2 bonded R1 of *ca.* 205 strands and *ca.* 135 strands per AuNP, respectively, and were selected for the following experiments.

### Optimization of the hairpin-locked swing arm (A)

The hairpin-locked A has two interrelated functions: a target recognizer for configuration switching and a free swing arm for grasping and releasing R when the hairpin is opened by a specific target. We optimized A with three important domains of **b^#^**, **b′**, and **e**, sequentially. Domain **b^#^** is designed to bind to the partial ATP aptamer in R and liberate R when the ATP/R complex is formed. To eliminate interference from the target recognizer, we designed a linear strand (W), which exposed domains **b^#^** and c* to R1 directly and to replace A to facilitate the optimization of domain **b^#^**. A series of W (W1 to W4) with increased complementary domain **b^#^** from 4 to 9 nt were employed to construct W/R-AuNP conjugates (Fig. 2c). Using the ME-induced Au–S bond dissociation reaction, the number of R1 tethered by different W on a single AuNP was calculated (Fig. 2d). W1 and W2 were bound to very small amounts of R1, which were consistent with the predictions at low *T*_m_ (30.1 °C for W1/R1 and 37.1 °C for W2/R1). The R1 amount of W4/R1-AuNP (*ca.* 214 strands) was greater than that of W3/R1-AuNP (*ca.* 99 strands), indicating that W4 had a stronger binding ability to R1 than W3. We further investigated the releasing process of R1 from W3 and W4 by incubating W/R-AuNP conjugates with 10 mM ATP for 1 h. It is observed that 84% of R1/W3 was bound to ATP, which was more than the 63.6% for R1/W4 (Fig. 2e). These results demonstrated that W3 with 7-nt domain **b^#^** exhibited better R1 releasing, while W4 with 9-nt domain **b^#^** displayed stronger R1 grasping. Next, we mixed W3 and W4 with S1 and S2, respectively, to assemble on AuNPs and incubated them with excess R1 to fabricate four kinds of nanomachines (S1/R1/W3-AuNP, S1/R1/W4-AuNP, S2/R1/W3-AuNP and S2/R1/W4-AuNP), to find the best combination of domain **b^#^** and domain **a** for the motion of nanomachines in response to ATP (Fig. 2f). We monitored the fluorescence increases in real-time within 60 min (Fig. S1a†). It is observed that the nanomachine of S2/R1/W3-AuNP exhibited the fastest fluorescence output. To evaluate the performance of these nanomachines more rationally, the rate constant *k* was calculated based on theoretical analysis. The whole reaction can be regarded as a first-order reaction when the ATP is present in a large excess (see the ESI†). As expected, by fitting the kinetic data to the first-order rate equation, the whole reaction of these nanomachines perfectly followed the first-order reaction with high correlation coefficients (Fig. S1b†). The rate constant *k* of S2/R1/W3-AuNP (4.2 × 10^–3^ ± 1.7 × 10^–4^ min^–1^) was 3.0 times and 5.8 times greater than that of S2/R1/W4-AuNP (1.4 × 10^–3^ ± 3.6 × 10^–5^ min^–1^) and S1/R1/W3-AuNP (7.2 × 10^–4^ ± 3.5 × 10^–5^ min^–1^), respectively (Fig. 1g). These findings demonstrate that 7-nt domain **b^#^** (W3) and 6-nt domain **a** (S2) were the best combination.

In design principle, A has to satisfy two requirements: one is high stability *via* the blocker (domain **b′**) to prevent self-opening of A in the presence of free R in the fabrication of the nanomachine, and the other is high specificity in recognizing the target *via* the loop of domain **e**. As a model, we employed a 22-nt DNA analog (T1) as the target, which has a sequence in common with miR-21, a well-known “oncomir” with overexpression in various cancers. The length of domain **b′** was designed to be 2 nt (A1), 4 nt (A2), 6 nt (A3) and 7 nt (A4), respectively, to sequester **b^#^** from binding R in the fabrication of the nanomachine (Fig. S2a†). The real-time fluorescence intensity of these nanomachines was measured (Fig. S2b†). A highest fluorescence intensity and a negligible fluorescence leakage of A2 with 4-nt domain **b′** were observed in the presence and absence of T1, respectively. Thus, A2 was selected to optimize domain **e**. Three kinds of A2 (12-nt domain **e**), A5 (11-nt domain **e**), and A6 (9-nt domain **e**) were tested using two targets (perfectly matched T1 and T2 with a single-base mutation to T1). As shown in Fig. S3,† A6 shows the best specificity in response to 5 nM T2 and also a good signal output. Using the optimized components of R1, S2 and A6 to fabricate the nanomachine, the motion is illustrated in detail in Fig. S4.†

### Characterization of the nanomachine *in vitro*

**Fig. 3:**
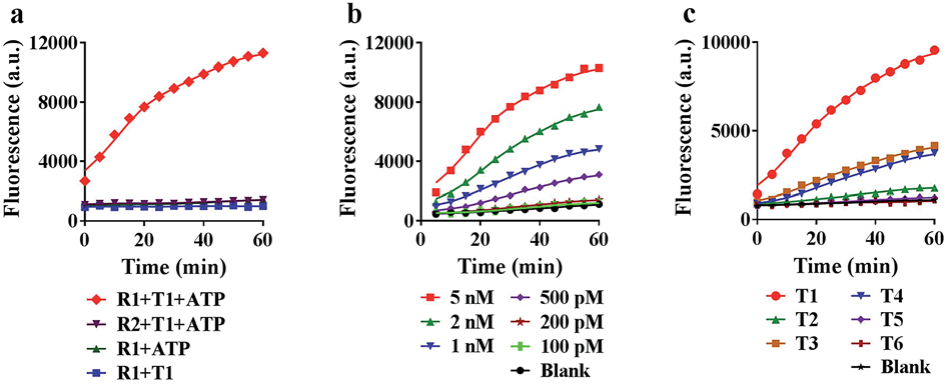
Characterization of the proposed nanomachine *in vitro*. (a) Real-time fluorescence curves of 0.1 nM R1 (S2/R1/A6-AuNP) and R2 (S2/R2/A6-AuNP) in response to 5 nM T1 in the presence of 5 mM ATP, and 0.1 nM R1 (S2/R1/A6-AuNP) in the absence of 5 mM ATP or 5 nM T1, respectively. (b) Operating curves of the 0.1 nM nanomachine (S2/R1/A6-AuNP) with 5 mM ATP in response to T1 at different concentrations (from 100 pM to 5 nM). (c) Operating curves of the 0.1 nM nanomachine (S2/R1/A6-AuNP) with 5 mM ATP in response to the perfectly matched target (T1) and five variants with mismatched bases (T2–T6). The experimental values represent the mean from three independent experiments.

To achieve the best performance *in vitro*, we optimized the concentrations of ATP and Mg^2+^ in the reaction system, and found that 5 mM ATP and 25 mM Mg^2+^ provided the highest fluorescence ratio (*F*/*F*_0_) (Fig. S5 and S6†). Under the optimized conditions, we further tested the motion of the nanomachine in the unfolding, grasping and releasing processes. As shown in Fig. 3a, the proposed nanomachine (S2/R1/A6-AuNP) operates well in the presence of T1 and ATP with a significant fluorescence output (red line). *Via* measuring the fluorescence increase every 1 min for the first 5 min, the initial rate was calculated to be 2.2 × 10^–10^ ± 1.4 × 10^–11^ mol min^–1^ (Fig. S7†). In contrast, no fluorescence change in the absence of ATP was observed because the nanomachine cannot operate without ATP as a fuel (blue line). It should be noted that, even if A6 was unfolded by T1 and grasped R1 from S2, the FAM in R1 was still greatly quenched by the AuNP. In the absence of T1, nearly no fluorescence change was observed (green line). The stability of the S/R duplex on AuNPs in the presence of ATP and the grasping process of the proposed nanomachine were also confirmed by non-denaturing polyacrylamide gel electrophoresis (PAGE) analysis (Fig. S8†). In addition, the nanomachine assembled with R2, which contains two mismatched bases on domain **c**, shows a slight fluorescence increase because the failure to grasp R2 hindered the autonomous motion of the swing arm (purple line).

Next, we investigated the sensitivity and selectivity of the proposed nanomachine. As shown in Fig. 3b, the fluorescence intensity gradually increased with an increase in the concentration of T1 from 100 pM to 5 nM. We obtained the calibration curves based on the fluorescence intensity *versus* T1 concentration. In the linear region from 100 pM to 2 nM, the regression equation is *F* = 3537.6[T] + 1056, with a correlation coefficient *R*^2^ of 0.9875 (Fig. S9†). To test the specificity of the proposed machine, we prepared five variants of single-base mismatches (T2–T4), a two-base mismatch (T5) and a DNA analogue (T6) of miR-141. It is observed that our nanomachine was able to differentiate the perfectly matched T1 from the single-base mismatch variants (Fig. 3c and S10†). This, again, further supports that 9-nt domain **e** in the loop of A6 allows good sequence specificity. In addition, the stability of our nanomachine was investigated by incubation with DNase I and HeLa cell lysis for 6 h, respectively. As expected, no significant fluorescence increase was observed compared to the background (Fig. S11†), indicating that DNA components assembled on AuNPs are highly resistant to enzymatic degradation due to the protection of AuNPs.

### Specific microRNA imaging in living cells

**Fig. 4:**
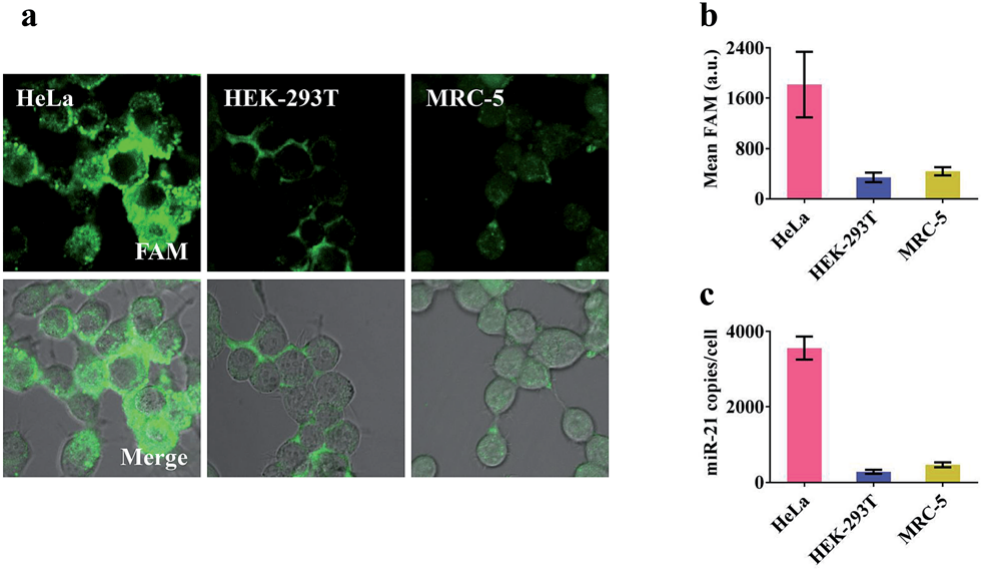
Analysis of miR-21 in living cells. (a) Confocal images of HeLa, HEK-293T and MRC-5 after treating with the 0.2 nM nanomachine (S2/R1/A6-AuNP) for 2 h. The merged image shows the mixture of the bright field and fluorescence of these cells. (b) Mean fluorescence values in each cell of the tested cell lines *via* analysis of NIS-elements. (c) qRT-PCR data of the amounts of miR-21 measured in the tested cells shown in (a). Error bars represent the standard deviation from three independent experiments.

Prior to intracellular experiments, the cytotoxicity of the proposed nanomachine was investigated by CCK-8 assay. After 2 h incubation, the viability of the cells was maintained at 95.9 ± 1.9% (Fig. S12†), confirming the good biocompatibility of AuNPs. Then we selected three types of cell lines, human cervical cancer cells (HeLa), human embryonic kidney cells (HEK-293T), and human embryonic lung fibroblast cells (MRC-5), which had different miR-21 expression profiles, to test the miRNA imaging capability of our developed nanomachine. As shown in Fig. S13,† the concentrations of ATP in these tested cells were calculated to be 4.2 mM in HeLa, 2.5 mM in HEK-293T, and 3.1 mM in MRC-5. For an approximate cell diameter of 13.9 μm,^31^ these concentrations were ample to power our nanomachine. After incubation of our proposed nanomachine with the tested cells for 2 h, the brightest fluorescence signal was observed in HeLa cells and was far greater than those in HEK-293T cells and MRC-5 cells (Fig. 4a). Fig. 4b shows that the quantized mean FAM intensity of HeLa cells was 5.3 times and 4.1 times greater than that of HEK-293T and MRC-5 cells, respectively. These observations were further confirmed by the data of qRT-PCR experiments (Fig. 4c). We noted that our nanomachines could distinguish the different miR-21 expression profiles of HEK-293T and MRC-5, and sensitively detect miR-21 with low expression in HEK-293T, demonstrating the good applicability and accuracy of our nanomachine for miRNA imaging.

### Investigation of specificity in living cells

**Fig. 5:**
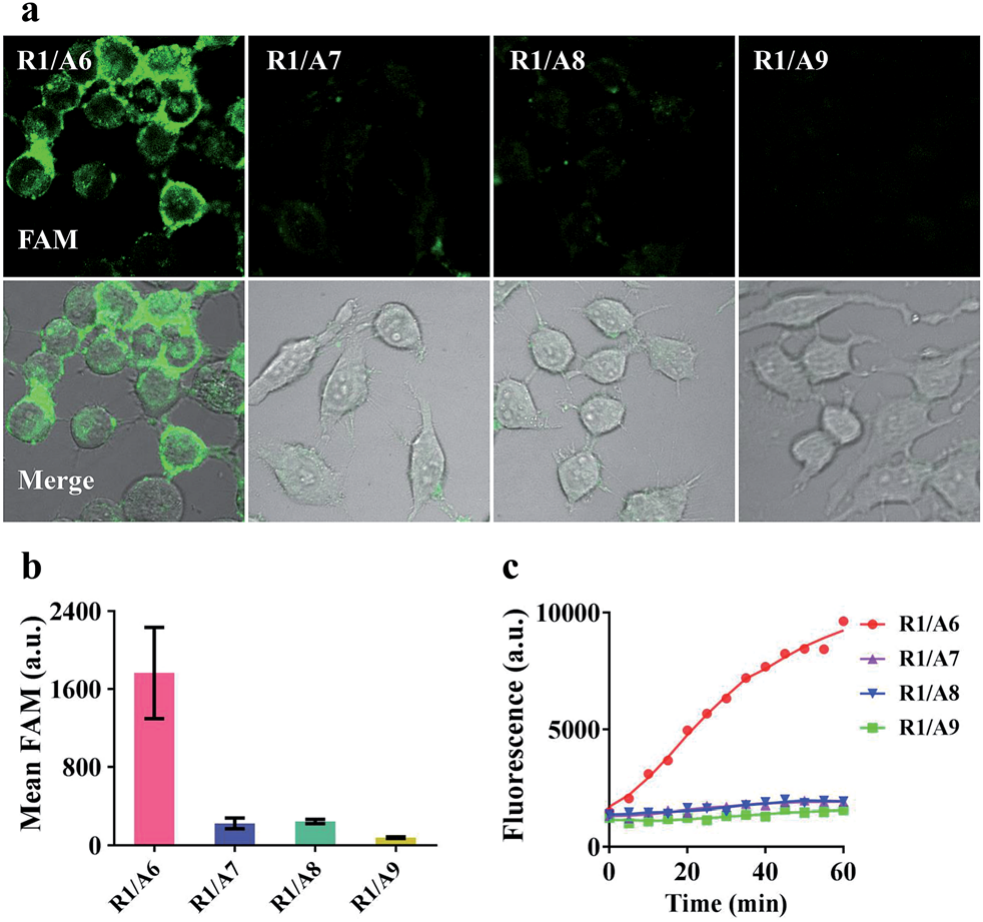
Specificity analysis of HeLa cells. (a) Confocal images of HeLa cells treated with different 0.2 nM nanomachines (S2/R1/A6-AuNP, S2/R1/A7-AuNP, S2/R1/A8-AuNP and S2/R1/A9-AuNP) for 2 h. (b) Mean fluorescence values of the tested nanomachines in HeLa cells *via* analysis of NIS-elements. Error bars represent the standard deviation from *n* = 3 experiments. (c) Real-time fluorescence intensity of different 0.1 nM nanomachines (S2/R1/A6-AuNP, S2/R1/A7-AuNP, S2/R1/A8-AuNP and S2/R1/A9-AuNP) in response to 5 nM T1.

To demonstrate that the operation of our nanomachine was specifically activated by the target miR-21, we fabricated a series of nanomachines assembled with A6, A7 and A8 with one- and two-mismatched bases on the recognition sequence of miR-21, respectively, and A9 with 9-nt polyT replacing domain **e** as control, and incubated these nanomachines with HeLa cells for 2 h. Only the nanomachine fabricated with A6 produced a significant fluorescence signal while A7 and A8 yielded negligible fluorescence, and nearly no fluorescence was observed in S2/R1/A9-AuNP (Fig. 5a and b). These results were consistent with the real-time monitoring *in vitro* (Fig. 5c), confirming the high specificity and stability of our nanomachine when applied to living cells.

### Real-time monitoring of the transfection and operation of the nanomachine in HeLa cells

**Fig. 6:**
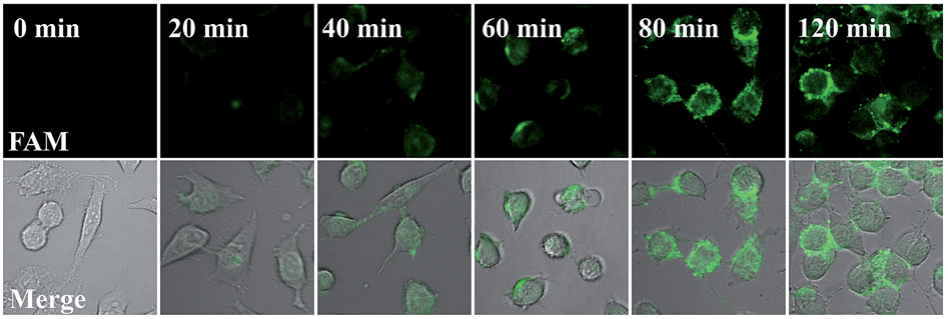
Real-time monitoring of the operation of the nanomachine in HeLa cells. HeLa cells were incubated with the 0.2 nM nanomachine (S2/R1/A6-AuNP) for 20 min, 40 min, 60 min, 80 min and 120 min, respectively, and collected to image immediately using a Nikon confocal scanning system. 0 min represents that the HeLa cells were not incubated with the nanomachine.

Our proposed nanomachine is designed with all reaction components assembled on the same AuNP, which means that the activation and autonomous operation of our nanomachine almost simultaneously follow its cellular uptake *via* the incubation procedure as long as a certain amount of the target miRNA is present in cells. To investigate the operation time of our developed nanomachine in living cells, we incubated HeLa cells with the 0.2 nM nanomachine and tracked the operation of the nanomachine in HeLa cells at different incubation time points. As shown in Fig. 6, a weak fluorescence was observed in the HeLa cells after 20 min, indicating that the nanomachine was successfully internalized into HeLa cells and activated by miR-21. The brightest fluorescence signal was achieved in about 80 min and no remarkable fluorescence increase was observed in 120 min, suggesting that the operation of the nanomachine proceeded to equilibrium with its intensity plateau in response to a certain amount of miR-21. Therefore, the total working time of our nanomachine *in vivo* including the transfection and operation time was about 80 min. In terms of the reaction time *in vivo*, our proposed nanomachine outperforms many machines reported previously (Table S2†). We attributed this to the enhanced local concentrations of all components assembled on a single AuNP, which is favourable for the rapid intramolecular reaction.

## Conclusions

In summary, we have developed a highly integrated DNA nanomachine powered by endogenous ATP to execute repetitive cycles of motion, and applied it for specific miRNA imaging inside living cells. Unlike other reported cellular DNA nanomachines with scattered multi-components and external fuel supply, we integrated a hairpin-trapped arm embedded with target recognition and a two-component track assembled with an ATP aptamer on a single AuNP, and extracted energy from the conformational transition of one track component upon binding ATP to drive the autonomous movement of the activated swing arm *in vivo*. All reaction components assembled on a single AuNP together not only favour the efficient cellular uptake of the entire system, but also exhibit a high degree control of the intramolecular reaction between the interacting components of the nanomachine, thus enhancing the speed of motion. This is of great importance for the autonomous movement of the machine in a complex cellular environment because the unpredictable distribution of exogenously delivered essential components would greatly impede the facile access to the interacting components of the machine, resulting in low productivity. Our developed DNA nanomachine represents a beautiful example of an elegant design of components, high integration mode, and the use of intracellular biomolecules as a driving force. We believe this work will lead to the development of other novel DNA nanomachines to execute diverse tasks inside living cells by customizing specific DNA components in response to intracellular stimuli.

## Conflicts of interest

There are no conflicts to declare.

## Supplementary Material

Supplementary information

## References

[cit1] Tyska M. J., Warshaw D. M. (2002). Cell Motil. Cytoskeleton.

[cit2] Vale R. D., Reese T. S., Sheetz M. P. (1985). Cell.

[cit3] Holzbaur E. L. F., Vallee R. B. (1994). Annu. Rev. Cell Biol..

[cit4] Hubscher U., Maga G., Spadari S. (2002). Annu. Rev. Biochem..

[cit5] Sherman W. B., Seeman N. C. (2004). Nano Lett..

[cit6] Shin J. S., Pierce N. A. (2004). J. Am. Chem. Soc..

[cit7] Kelly T. R. (2005). Angew. Chem., Int. Ed..

[cit8] Omabegho T., Sha R., Seeman N. C. (2009). Science.

[cit9] Jung C., Allen P. B., Ellington A. D. (2016). Nat. Nanotechnol..

[cit10] Qu X., Zhu D., Yao G., Su S., Chao J., Liu H., Zuo X., Wang L., Shi J., Wang L., Huang W., Pei H., Fan C. (2017). Angew. Chem., Int. Ed..

[cit11] Zhang H., Lai M., Zuehlke A., Peng H., Li X. F., Le X. C. (2015). Angew. Chem., Int. Ed..

[cit12] Yang X., Tang Y., Mason S. D., Chen J., Li F. (2016). ACS Nano.

[cit13] Yehl K., Mugler A., Vivek S., Liu Y., Zhang Y., Fan M., Weeks E. R., Salaita K. (2016). Nat. Nanotechnol..

[cit14] Yurke B., Turberfield A. J., Mills Jr A. P., Simmel F. C., Neumann J. L. (2000). Nature.

[cit15] Tian Y., Mao C. (2004). J. Am. Chem. Soc..

[cit16] Wang C., Huang Z., Lin Y., Ren J., Qu X. (2010). Adv. Mater..

[cit17] Lund K., Manzo A. J., Dabby N., Michelotti N., Johnson-Buck A., Nangreave J., Taylor S., Pei R., Stojanovic M. N., Walter N. G., Winfree E., Yan H. (2010). Nature.

[cit18] Muscat R. A., Bath J., Turberfield A. J. (2011). Nano Lett..

[cit19] Douglas S. M., Bachelet I., Church G. M. (2012). Science.

[cit20] Thubagere A. J., Li W., Johnson R. F., Chen Z., Doroudi S., Lee Y. L., Izatt G., Wittman S., Srinivas N., Woods D., Winfree E., Qian L. (2017). Science.

[cit21] Wickham S. F., Endo M., Katsuda Y., Hidaka K., Bath J., Sugiyama H., Turberfield A. J. (2011). Nat. Nanotechnol..

[cit22] Liang C. P., Ma P. Q., Liu H., Guo X. G., Yin B. C., Ye B. C. (2017). Angew. Chem., Int. Ed..

[cit23] Li D., Zhou W., Yuan R., Xiang Y. (2017). Anal. Chem..

[cit24] Modi S., M. G S., Goswami D., Gupta G. D., Mayor S., Krishnan Y. (2009). Nat. Nanotechnol..

[cit25] Modi S., Nizak C., Surana S., Halder S., Krishnan Y. (2013). Nat. Nanotechnol..

[cit26] Saha S., Prakash V., Halder S., Chakraborty K., Krishnan Y. (2015). Nat. Nanotechnol..

[cit27] Peng H., Li X. F., Zhang H., Le X. C. (2017). Nat. Commun..

[cit28] Zhou M., Liang X., Mochizuki T., Asanuma H. (2010). Angew. Chem., Int. Ed..

[cit29] Wen Y., Xu L., Wang W., Wang D., Du H., Zhang X. (2012). Nanoscale.

[cit30] Yang Y., Goetzfried M. A., Hidaka K., You M., Tan W., Sugiyama H., Endo M. (2015). Nano Lett..

[cit31] Mateus A., Matsson P., Artursson P. (2013). Mol. Pharmaceutics.

