# Supplementary material for: A highly integrated DNA nanomachine operating in living cells powered by an endogenous stimulus
Source: Chem Sci. 2018 Feb 23;9(13):3299–304. doi: 10.1039/c8sc00049b (PMC5931192; doi:10.1039/c8sc00049b)
Supplement: Supplementary file 1 [file SC-009-C8SC00049B-s001.pdf]

Electronic Supplementary Material (ESI) for Chemical Science.
This journal is © The Royal Society of Chemistry 2018

SUPPORTING INFORMATION

A Highly Integrated DNA Nanomachine Operating in Living Cells Powered by an Endogenous Stimulus

Pei-Qiang Ma,[ab] Cheng-Pin Liang,[b] He-Hua Zhang,[b] Bin-Cheng Yin,[*ab] and Bang-Ce Ye[ab]

[a] Collaborative Innovation Center of Yangtze River Delta Region Green Pharmaceuticals, College of Pharmaceutical Sciences, Zhejiang University of Technology, Hangzhou 310014, Zhejiang, China

[b] Lab of Biosystem and Microanalysis, State Key Laboratory of Bioreactor Engineering, East China University of Science and Technology, Shanghai, 200237, China

*Corresponding author: Bin-Cheng Yin, Email: binchengyin@ecust.edu.cn

Table of contents

Experimental sections P2-P7

Table S1. Sequence information for oligonucleotides and miRNAs used in this study. P2-P3

Supporting figures and table

Figure S1. Optimization of the domain $b^{\#}$ of W. P7

Figure S2. Optimization of the domain b' of A. P8

Figure S3. Optimization of the domain e of A. P9

Figure S4. Detailed sequence information for the operation of the proposed DNA nanomachine in response to target. P10

Figure S5. Effect of ATP concentration on the operation of the nanomachine. P11

Figure S6. Effect of Mg^{2+} concentration on the operation of the nanomachine. P11

Figure S7. Calculation of initial rate of the nanomachine. P12

Figure S8. Non-denaturing PAGE analysis. P12

Figure S9. Sensitivity investigation of the nanomachine. P13

Figure S10. Specificity investigation of the nanomachine. P13

Figure S11. Stability investigation of the nanomachine. P13-P14

Figure S12. Cytotoxicity analysis of nanomachine in HeLa cells. P14

Figure S13. Measurement of ATP amount in a single cell. P14

Table S2. Comparison of the needed incubation time for our method and other methods for the miRNA imaging. P14-P15

References P15

Experimental section

Reagents and materials. The oligonucleotides were synthesized and purified using HPLC by Sangon Biological Engineering Technology & Services Co., Ltd. (Shanghai, China). miRNAs were synthesized and purified using HPLC by Takara Biotechnology Co. Ltd. (Dalian, China). The detailed sequence of these oligonucleotides and miRNAs are listed in Table S1. Hydrogen tetrachloroaurate (III) trihydrate ($HAuCl_4 \cdot 3H_2O$) was purchased from Alfa Aesar (China). Sodium citrate, sodium dodecyl sulfate (SDS), tris(hydroxymethyl)aminomethane (Tris) and tris(2-carboxyethyl) phosphine (TCEP) were purchased from Sigma-Aldrich, Inc. (Saint Louis, MO, USA). Dithiothreitol (DTT) was purchased from Aladdin Chemistry Co. Ltd. (Shanghai, China). 6-Mercapto-1-hexanol (MCH, 98%) was purchased from TCI Inc. (Shanghai, China). Dibasic sodium phosphate (Na_2HPO_4), sodium dihydrogen phosphate trihydrate ($NaH_2PO_4 \cdot 3H_2O$), and 37% hydrochloric acid (HCl) were purchased from Lingfeng Chemical Reagent Co., Ltd. (Shanghai, China). 10×phosphate buffered saline (10×PBS, pH 7.4, containing 1370 mM sodium chloride, 27 mM potassium chloride, 101 mM dibasic sodium phosphate, and 18 mM monobasic potassium phosphate) was purchased from Shanghai Double-Helix biotech. Co. Ltd. (China). ATP bioluminescent assay kit was purchased from Beyotime Biotechnology Co. Ltd. (Shanghai, China). NCode™ EXPRESS SYBR® GreenER™ miRNA qRT-PCR Kit was purchased from Invitrogen (Grand Island, NY, USA). Cell lines, including a human cervical cancer cell line (HeLa), a human embryonic kidney cell line (HEK-293T), and a human embryonic lung fibroblast cell line (MRC-5) were obtained from the Cell Bank of Type Culture Collection of the Chinese Academy of Sciences (Shanghai, China). Dulbecco's modified Eagle's medium (DMEM), Opti-MEM, fetal bovine serum (FBS), and trypsin/EDTA (0.25%) were purchased from Gibco (Thermo Fisher Scientific, Gaithersburg, USA). CCK-8 reagent was purchased from Dojindo Laboratories (Kumamoto, Japan). RIPA lysis buffer was purchased from Solarbio Science and Technology Co., Ltd (Beijing, China).

Table S1. **Sequence information for oligonucleotides and miRNAs used in this study**

Name	[a] Sequence (5′-3′)
S1 (4-nt a)	SH-TTTTTTTTTAACCTCCGCTTTTCTCCCCCAGGT
S2 (6-nt a)	SH-TTTTTTTTTAACCTCCGCTTTTTTCCCCCAGGT
S3 (8-nt a)	SH-TTTTTTTTTAACCTCCGCTTTTTTTTCCCAGGT
S4 (10-nt a)	SH-TTTTTTTTTAACCTCCGCTTTTTTTTTTCAGGT
R1	AGCTAACCTGGGGGAGTATTGCGGAGGAAGGTTT-FAM
R2	A*T*CT*T*ACCTGGGGGAGTATTGCGGAGGAAGGTTT-FAM
R*	ACCTTCCTCCGCAATACTCCCCCAGGTTAGCT
W1 (4-nt b[#])	AGGTTAGCTTATCATTTTTTTTTTTTTTTTTTTTTTTT-SH
W2 (5-nt b[#])	CAGGTTAGCTTATCATTTTTTTTTTTTTTTTTTTTTTTT-SH
W3 (7-nt b[#])	CCCAGGTTAGCTTATCATTTTTTTTTTTTTTTTTTTTTTTT-SH
W4 (9-nt b[#])	CCCCCAGGTTAGCTTATCATTTTTTTTTTTTTTTTTTTTTTTT-SH
A1 (2-nt b')	CCCAGGTTAGCTTATCA-T_{10}-TCAACATCAGTCTGATAAGCTATTTTTGG-T_{15}-SH
A2 (4-nt b')	CCCAGGTTAGCTTATCA-T_{10}-TCAACATCAGTCTGATAAGCTATTTTGGG-T_{15}-SH
A3 (6-nt b')	CCCAGGTTAGCTTATCA-T_{10}-TCAACATCAGTCTGATAAGCTATCCTGGG-T_{15}-SH

A4 (7-nt b')	CCCAGGTTAGCTTATCA-T_{10}-TCAACATCAGTCTGATAAGCTAACCTGGG-T_{15}-SH
A5 (11-nt e)	CCCAGGTTAGCTTATCAG-T_{10}-TCAACATCAGTCTGATAAGCTATTTTGGG-T_{15}-SH
A6 (9-nt e)	CCCAGGTTAGCTTATCAGAC-T_{10}-TCAACATCAGTCTGATAAGCTATTTTGGG-T_{15}-SH
A7 (mis-2 nt)	CCCAGGTTAGCTTATCAAAC-T_{10}-TCAACATTAGTTTGATAAGCTATTTTGGG-T_{15}-SH
A8 (mis-4 nt)	CCCAGGTTAGCTAATCAAAC-T_{10}-TCATCATTAGTTTGATTAGCTATTTTGGG-T_{15}-SH
A9 (polyT)	CCCAGGTTAGCTAATCAAAC-T_{10}-TTTTTTTTTGTTTGATTAGCTATTTTGGG-T_{15}-SH
miR-21	UAGCUUAUCAGACUGAUGUUGA
T1	TAGCTTATCAGACTGATGTTGA
T2	TAGCTTATCACACTGATGTTGA
T3	TAGCTAATCAGACTGATGTTGA
T4	TAGCTTATCAGACTGTTGTTGA
T5	TAACTGATCAGACTGATGTTGA
T6	TAACACTGTCTGGTAAAGATGG

[a] Green characters represent the mismatched bases between domain a of A and domain a' of R. Blue characters represent domain b of R, domain b* of S, and domains b$^{\#}$ and b' of A. Rose red characters represent domain d of S and domain d* of R. Orange characters represent domains c of R, and domains c* and c' of A. Italics characters represent different bases compared with R in domain c. Purple characters represent domain e of A. Red characters represent the sequence of the target (T). Underlined characters represent ATP aptamer sequence in R. Double underlined characters in A7, A8 and A9 represent different bases compared with A6. Framed characters represent different bases compared with T1 in the tested target.

Instrumentation. The fluorescence emission signal and ultraviolet visible (UV-vis) absorbance were recorded by a microplate reader (BioTek Instruments, Inc., Winooski, Vermont, USA) using a black 384-well microplate and a transparent 384-well microplate, respectively. For FAM detection, the excitation and emission wavelengths were set at 485 nm and 520 nm, respectively. The cells were cultured in a cell incubator (Eppendorf Galaxy 170S, Germany). The cells were visualized under a Nikon confocal scanning system with 24-well cell culture plate with bottom glass (In Vitro Scientific). The qRT-PCR data were collected with a BIO-RAD CFX connect TM Real-Time system (Singapore)

Fabrication of DNA nanomachine. The nanomachines were prepared using 20 nm AuNPs, which were synthesized according to our previous work[1]. The prepared 20 nm AuNPs solution was concentrated to 10 nM by centrifugation at 12 000 rpm for 10 min and stabilized by capping with SDS by adding 0.1% SDS (wt %). Before mixing with as-prepared AuNPs, 20 μM substrate strand (S) and 20 μM hairpin-locked swing arm (A or W) were incubated with 5 mM TCEP for 15 min, respectively. An aliquot of 400 μL mixture containing 10 mM phosphate buffer (pH 7.5), 6 μL A (or W (20 μM)) and 40 μL S (20 μM), or only 40 μL W (20 μM) with 80 μL as-prepared AuNPs (10 nM) (S/R/A (or S/R/W)-AuNP or R/W-AuNP) was incubated at room temperature for 15 min. After that, 400 μL NaCl solution (2.0 M) was added dropwise to the above mixture with vigorous vortexing and incubated for 2 h. Then, the mixture was centrifuged at 13 000 rpm for 15 min. The excess A (or W) and S or W were removed in the supernatant. The precipitated A (or W) and S or W functionalized AuNPs (S/A-AuNPs (or S/W-AuNPs) or W-AuNP) were resuspended in 800 μL 1×PBS (pH 7.4), and mixed with 12 μL 6-Mercapto-1-hexanol (MCH) (100 μM). After 1 h incubation, the AuNP conjugate solution was centrifuged and washed three times with 1×PBS containing 0.05 % Tween-20. The precipitated AuNP conjugates were resuspended in 800 μL 1×PBS, containing 40 μL R (20 μM), 20 μL $MgCl_2$ (100 mM) and 20 μL NaCl

(2 M), heated to 60°C for 10 min, and slowly cooled to room temperature followed by overnight incubation at room temperature. Finally, the resultant AuNP conjugates (S/R/A (or S/R/W)-AuNPs) was washed five times with 1×PBS containing 0.05 % Tween-20 and stored in 1×PBS.

Calculation of the numbers of R tethered by S or W. To measure the tethered number of R by S or W on a single AuNP, 100 μL solution containing 0.1 nM nanomachine (S/R/A-AuNP or R/W-AuNP) was mixed with 20 mM 2-mercaptoethanol (ME) in 200 mM Tris-HCl, 25 mM Mg^{2+} and 10 mM ATP, and then incubated overnight at room temperature. Then the solution was centrifuged at 13 000 rpm for 15 min at 25 °C to precipitate the AuNPs and the supernatant (~95 μL) was transferred onto a 384-well black plate for fluorescence detection. The molar concentration of the released R was determined using a standard curve for the fluorescence intensity *versus* different concentrations of R. Then the average number of R per AuNP could be calculated from the concentration of AuNPs.

Calculation of the rate constant (k) for the whole reaction. The overall operation of the nanomachine fabricated with A or W can be expressed according to the following reactions.

Unfolding reaction: $A + T \to A/T$

Grasping reaction: $S/R + A/T \to S + A/T/R$ ($S/R + W \to S + W/R$)

Releasing reaction: $A/T/R + ATP \to A/T + ATP/R$ ($W/R + ATP \to W + ATP/R$)

The whole reaction of the nanomachine can be expressed as follows,

$$S/R + ATP \xrightarrow{A/T} S + ATP/R \ (S/R + ATP \xrightarrow{W} S + ATP/R)$$

When 10 mM ATP was chosen as a working concentration for optimizing the components of the proposed nanomachine (in large excess compared to the amount of S/R), the entire reaction can be viewed as pseudo-first order, and the kinetics can be fit to the first-order rate equation:

$$\ln([S/R]_t) = -kt + \ln([S/R]_0) = \ln([S/R]_0 - [ATP/R]_t)$$

where $[S/R]_t$ is the concentration of S/R at time t, $[S/R]_o$ is the initial concentration of reactant S/R, $[ATP/R]_t$ is the concentration of reactant ATP/R at time t, $[S/R]_o$ can be calculated from the number of S/R on a single AuNP, $[ATP/R]_t$ can be calculated by the regression curve of fluorescence intensity *versus* $[ATP/R]$. Therefore, we fitted the dynamic data to the first-order rate equation and the reciprocal of the slope is the rate constant k.

Optimization of functional domains of W, S and A. To optimize the domain $b^{\#}$ of W, we replaced A with a linear strand W to fabricate the nanomachines. To observe the influence of different length of $b^{\#}$ on the grasp and the releasing, different W were assembled on AuNP without S, and incubated with R for fabricating R/W-AuNP. The fluorescence intensity of 0.1 nM nanomachines (R1/W1-AuNP, R1/W2-AuNP, R1/W3-AuNP and R1/W4-AuNP) were incubated with 10 mM ATP and 20 mM ME for 1 h and 12 h.

A series of nanomachines was prepared by using W1, W2, W3, and W4 with S2 or S3 (S2/R1/W1-AuNP, S2/R1/W2-AuNP, S2/R1/W3-AuNP, S2/R1/W4-AuNP, S3/R1/W1-AuNP, S3/R1/W2-AuNP, S3/R1/W3-AuNP and S3/R1/W4-AuNP). The real-time fluorescence intensity was measured for 100 μL reaction system containing 0.1 nM prepared nanomachines, 10 mM ATP, 200 mM Tris-HCl (pH 7.6) and 25 mM $MgCl_2$.

To investigate the block efficiency of A by domain b', a series of nanomachines was prepared using A1, A2, A3 and A4 (S2/R1/A1-AuNP, S2/R1/A2-AuNP, S2/R1/A3-AuNP and S2/R1/A4-AuNP). Real-time fluorescence intensity was measured for 100 μL reaction system containing 0.1 nM prepared nanomachines, 5 nM T1, 10 mM ATP, 200 mM Tris-HCl (pH 7.6) and 25 mM $MgCl_2$. The blank samples were prepared in the same way but without the addition of T1.

To optimize the domain e on A, a series of nanomachines was prepared using A1, A5, and A6 (S2/R1/A1-AuNP, S2/R1/A5-AuNP and S2/R1/A6-AuNP) in response to T1 or T2, respectively. Real-time fluorescence intensity was measured for 100 μL reaction system containing 0.1 nM prepared nanomachines, 5 nM T1 (or T2), 10 mM ATP, 200 mM Tris-HCl (pH 7.6) and 25 mM $MgCl_2$. The blank samples were prepared in the same way but without the addition of T1 (or T2).

Optimization of reaction conditions for the nanomachine *in vitro*. To obtain the optimal operation of the proposed nanomachine, we optimized two parameters of reaction conditions, the concentrations of ATP and Mg^{2+}. For the optimization of the concentration of ATP, a series of 0.1 nM nanomachines (S2/R1/A6-AuNP) was mixed with 5 nM T1 and ATP at different concentrations (0, 2.5, 5, 7.5 and 10 mM) in 100 μL solution of 200 mM Tris-HCl (pH 7.6) and 25 mM $MgCl_2$. The real-time florescence intensities of the prepared mixtures were monitored for 1 h. The blank samples were treated in the same way but without the addition of T1.

For the optimization of the concentration of Mg^{2+}, a series of 0.1 nM nanomachines (S2/R1/A6-AuNP) was mixed with 5 nM T1, 5 mM ATP, and $MgCl_2$ at different concentrations (0, 25, 50, 75 and 100 mM) in 100 μL solution of 200 mM Tris-HCl (pH 7.6). The real-time florescence intensities of the prepared mixtures were monitored for 1 h. The blank samples were treated in the same way but without the addition of T1.

Gel electrophoresis analysis to test the feasibility of the proposed nanomachine. A volume of 100 μL reaction mixture containing 2 nM S2/R1/A6-AuNP, 400 nM T1, or 10 mM ATP or 500 nM R* in 200 mM Tris-HCl with 25 mM $MgCl_2$, was incubated at room temperature for 60 min. After incubation, the mixture was centrifuged at 13 000 rpm for 15 min to precipitate AuNPs and the supernatant was collected for 20% non-denaturing polyacrylamide gel (PAGE). The PAGE experiments were carried out according to our previous work[1].

Investigation of the sensitivity and specificity of the nanomachine *in vitro*. To investigate the sensitivity of the nanomachine, a series of 0.1 nM nanomachines (S2/R1/A6-AuNP) was mixed with 5 mM ATP and T1 at different concentrations (from 100 pM to 5 nM) in 100 μL solution containing 200 mM Tris-HCl (pH 7.6) and 25 mM $MgCl_2$. Then, the real-time florescence intensities of the prepared mixtures were monitored for 1 h. The blank samples were treated in the same way but without the addition of T1.

To investigate the specificity of the nanomachine, a series of 0.1 nM nanomachines (S2/R1/A6-AuNP) was mixed with 5 mM

ATP and 5 nM different targets (T1-T6) in 100 μL solution containing 200 mM Tris-HCl (pH 7.6) and 25 mM $MgCl_2$. Then, the real-time florescence intensities of the prepared mixtures were monitored for 1 h. The blank samples were treated in the same way but without target addition.

Investigation of the stability of the nanomachine. To investigate the stability of the nanomachine in DNase I and cell lysis, 0.1 nM nanomachines (S2/R1/A6-AuNP) in 1×PBS was mixed with 50 U/L DNase I or 50% (volume ratio) HeLa cell lysis for 6 h. HeLa cells were lysed using RIPA lysis buffer.

Cell culture. Three cell lines (HeLa, HEK-293T and MRC-5) were cultured in 25 cm^2 cell culture flask containing 5 ml DMEM, supplemented with 10% fetal bovine serum (FBS) and 100 U/mL penicillin-streptomycin, and maintained in a humidified atmosphere of 5% CO_2 at 37 ^oC.

Investigation of the cytotoxicity of the nanomachine in living cells with the CCK-8 assay. Using HeLa cells as model, HeLa cells were seeded into a 96-well cell culture plate (Corning, USA) with 1×10^4 cells per well and incubated for 24 h. After that, cells were washed three times with 1×PBS. An aliquot of 100 μL Opti-MEM containing 0.2 nM nanomachines was added to every well of the treatment group of HeLa cells. The well in the control group of HeLa cells was treated with 100 μL Opti-MEM without 0.2 nM the nanomachine. Then, the plate was incubated in a humidified incubator containing 5% CO_2 at 37 ^oC. At different time points (1, 2, 3, 4 and 6 h), an aliquot of 10 μL CCK-8 reagent was added to the above treatment and control groups, respectively, and incubated for another 1 h, and the OD_{450} was measured to obtain A_t and A_c. In addition, blank samples were prepared as follows. Two clear wells without cells were treated with 100 μL Opti-MEM with and without 0.2 nM nanomachines, respectively, and then an aliquot of 10 μL CCK-8 reagent was added to the above wells, and incubated in a humidified incubator containing 5% CO_2 at 37 ^oC for 1 h. After that, the OD_{450} of these two wells were obtained as $A_{blank(t)}$ and $A_{blank(c)}$, respectively. The cell viability at different time points (1, 2, 3, 4 and 6 h) can be calculated based on the following formula. The cytotoxicity experiments were repeated three times.

$$(A_t - A_{blank(t)})/(A_c - A_{blank(c)})\times 100 = cell\ viability\ (\%)$$

Calculation of the concentration of ATP in tested cell lines. An ATP bioluminescent assay kit was used for calculating the concentration of ATP in HeLa, MRC-5 and HEK-293T cells. The three kinds of cells were seeded into a 6-well plate with 2.5 mL DMEM, supplemented with 10% fetal bovine serum (FBS) and 100 U/mL penicillin-streptomycin, and maintained in a humidified atmosphere of 5% CO_2 at 37 ^oC for 24 h. Then, cells were washed three times with 1×PBS and counted with a hemocytometer. After that, we determined the molar quantities of ATP according to the manufacturer's instructions. At last, the concentration of ATP in a single cell were calculated from the quantity and the volume of cells[2].

MiRNA imaging in living cells. The procedures for miRNA imaging using a Nikon confocal scanning system were as follows. HeLa, HEK-293T, and MRC-5 (1×10^5 cells per well) were seeded into a 24-well plate with 500 μL DMEM, supplemented with 10% fetal bovine serum (FBS) and 100 U/mL penicillin-streptomycin, and maintained in a humidified atmosphere of 5% CO_2 at 37 ºC for 24 h. After that, cells were washed three times with 1×PBS. An aliquot of 500 μL Opti-MEM containing 0.2 nM nanomachines was added to every well of treatment group. After incubation for another 2 h, the cells were washed three times

with 1×PBS and captured using a Nikon confocal scanning system with a 40× objective. All imaging experiments were repeated three times. The mean FAM intensity in a single cell was analyzed using NIS element software.

Investigation of specificity in living cells. To investigate the specificity of our nanomachine in living cells. Four kinds of nanomachines (S2/R1/A6-AuNP, S2/R1/A7-AuNP, S2/R1/A8-AuNP and S2/R1/A9-AuNP) were assembled. Using HeLa cells as a model, the procedures for confocal imaging were similar to the miRNA imaging except for that the cells were treated with different nanomachines (S2/R1/A6-AuNP, S2/R1/A7-AuNP, S2/R1/A8-AuNP and S2/R1/A9-AuNP). The mean FAM intensity in a single cell was analyzed using NIS element software. The real-time florescence analysis *in vitro* were monitored using the optimal conditions (0.1 nM nanomachine, 200 mM Tris-HCl (pH 7.6), 5 mM ATP, 25 mM Mg^{2+} and 5 nM T1).

qRT-PCR experiment. For qRT-PCR experiments, HeLa, HEK-293T and MRC-5 were cultured in 75cm^2 cell culture flasks containing 15 mL DMEM, supplemented with 10% fetal bovine serum (FBS) and 100 U/mL penicillin-streptomycin, and maintained in a humidified atmosphere of 5% CO_2 at 37 ℃. The cells were digested from the flask by trypsin, washed three times with 1×PBS, and counted with a hemocytometer. The cell lysates were prepared according our previous work[3]. Quantitative polymerase chain reaction (qPCR) was employed for the quantification of miRNA using the qRT-PCR Kit in a BIO-RAD CFX connect TM Real-Time system. Experiments were repeated three times, and data analysis was performed by 7500 Software v2.3.

Supporting figures and table

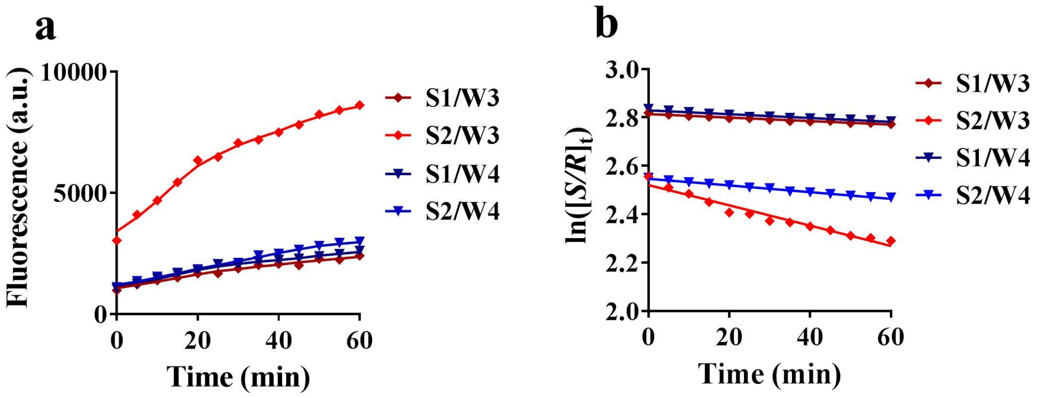

Fig. S1 Optimization of the domain b# of W. (a) Real-time fluorescence intensity was measured from 100 μL reaction system containing 0.1 nM nanomachines (S1/R1/W3-AuNP, S2/R1/W3-AuNP, S1/R1/W4-AuNP or S2/R1/W4-AuNP), 10 mM ATP and 25 mM Mg^{2+}, respectively. (b) Calculation of the rate constant k by plotting $\ln([S/R]_t)$ (equal to $\ln([S/R]_0 - [ATP/R]_t)$) as a function time and fitting the data using least-squares linear regression.

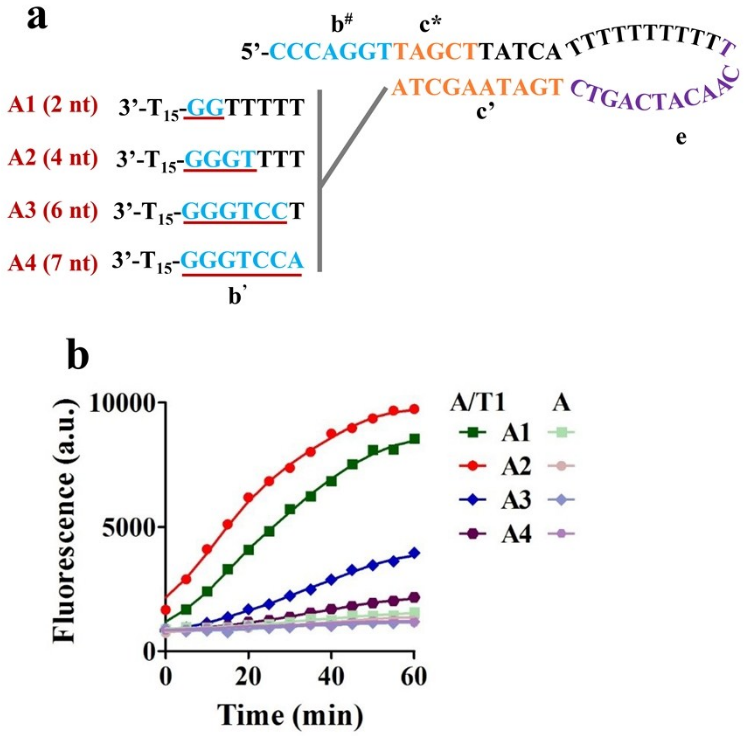

Fig. S2 Optimization of the domain b' of A. (a) Detailed sequences of A1-A4 with different domain b' lengths. (b) Real-time fluorescence intensity was measured from 100 μL reaction system containing 0.1 nM nanomachines (S2/R1/A1-AuNP, S2/R1/A2-AuNP, S2/R1/A3-AuNP or S2/R1/A4-AuNP), 10 mM ATP and 25 mM Mg^{2+} in the presence and absence of 5 nM T1, respectively.

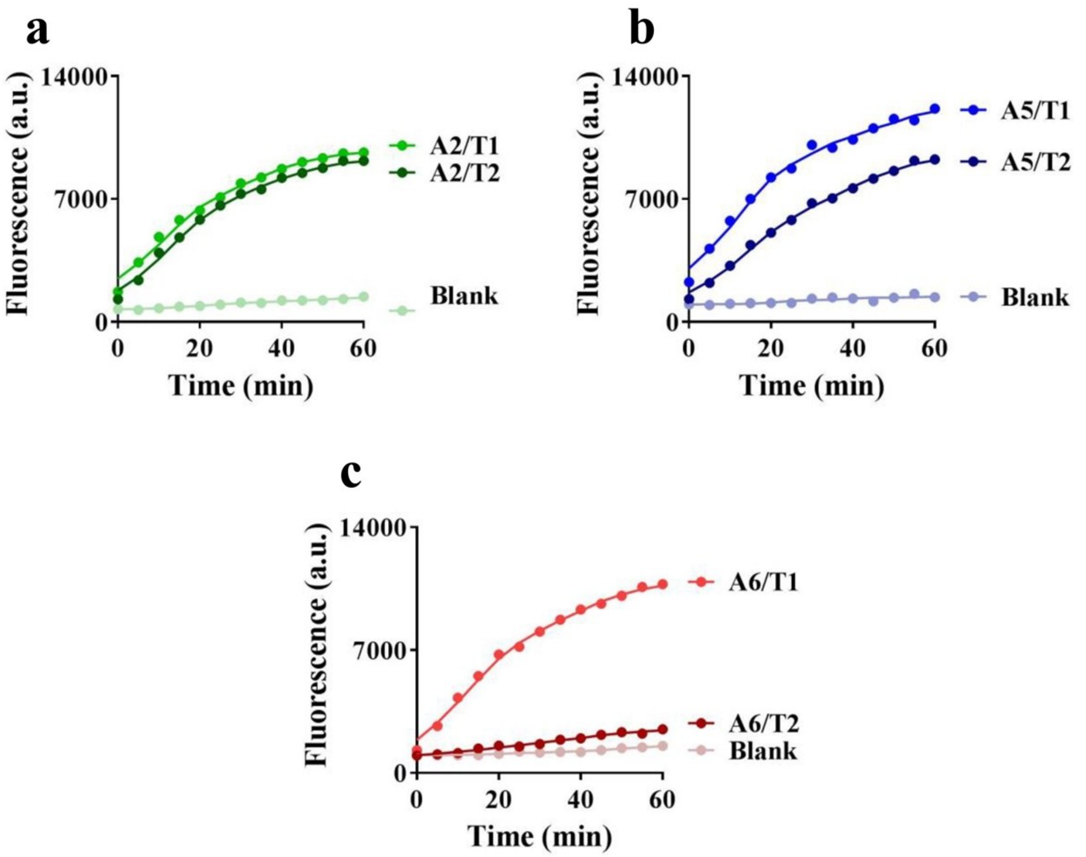

Fig. S3 Optimization of the domain e of A. Real-time fluorescence intensity monitored for three types of nanomachines of (a) S2/R1/A2-AuNP, (b) S2/R1/A5-AuNP and (c) S2/R1/A6-AuNP in response to 5 nM T1 and T2. The concentration of nanomachine was 0.1 nM and the reaction system contained 10 mM ATP.

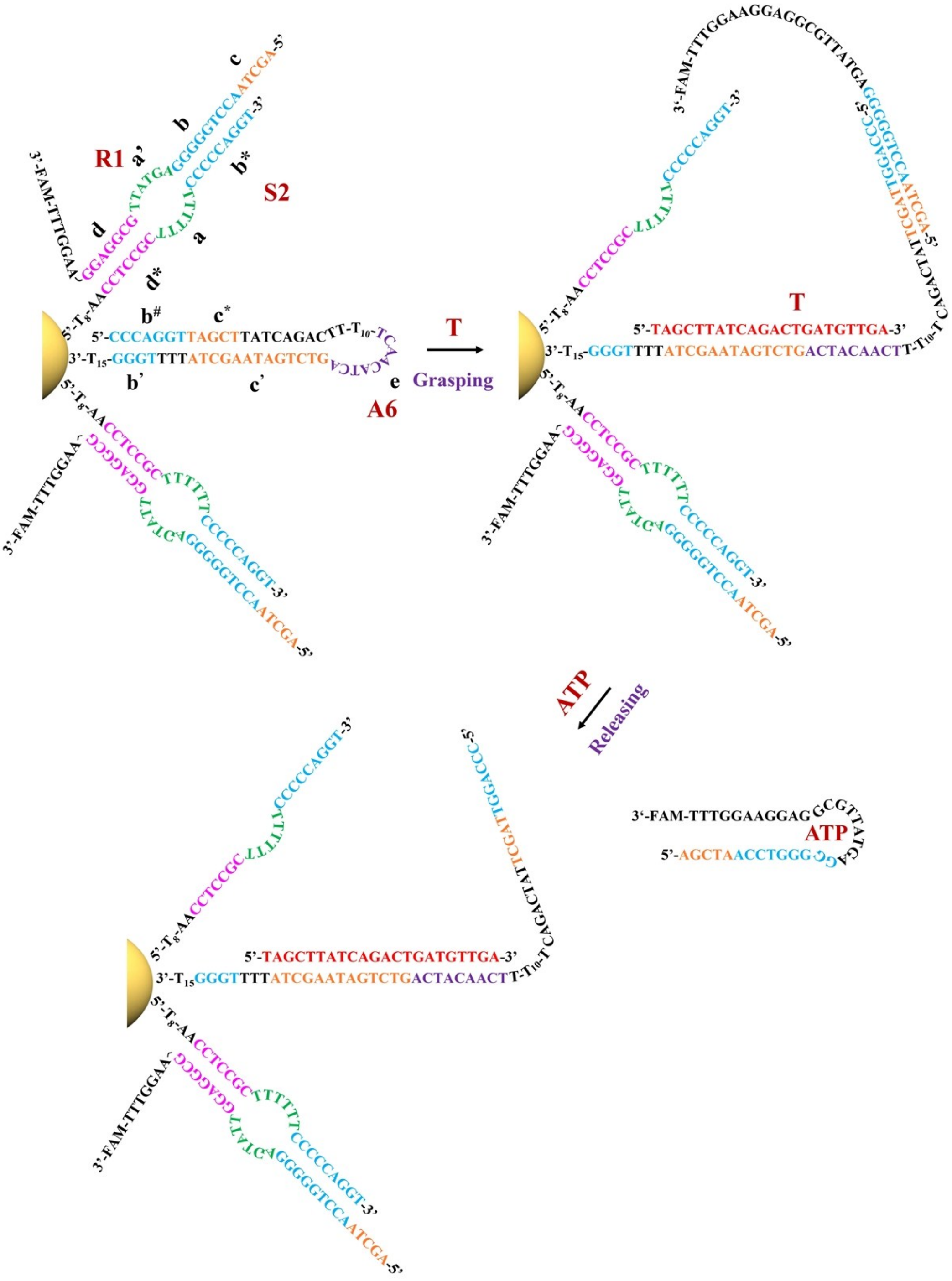

Fig. S4 Detailed sequence information for the operation of the proposed DNA nanomachine in response to specific target.

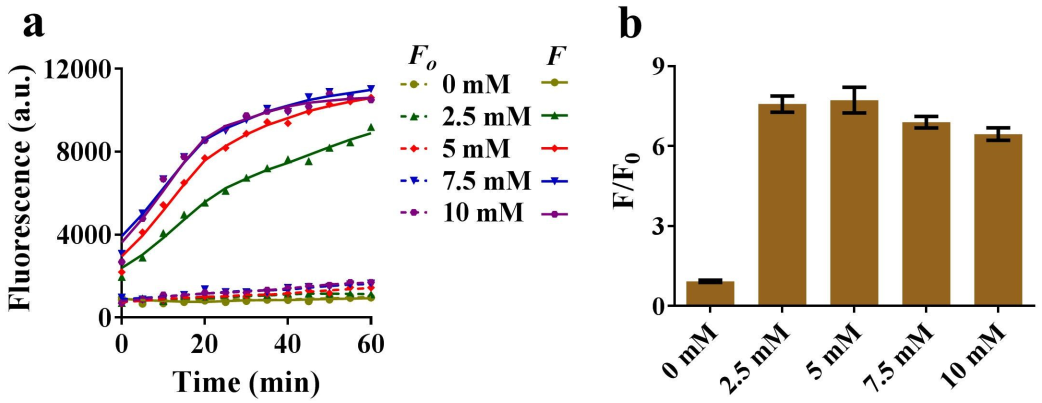

Fig. S5 Effect of ATP concentration on the operation of the nanomachine. (a) Real-time fluorescence intensity was measured for 100 μL reaction system containing 0.1 nM nanomachine (S2/R1/A6-AuNP), 25 mM Mg^{2+} and ATP at different concentrations (0, 2.5 mM, 5 mM, 7.5 mM and 10 mM). The solid and dashed lines represent fluorescence intensity in the presence and absence of 5 nM T1, respectively. (b) Fluorescence ratios (F/F_0) derived from the data (A), where F and F_0 are the fluorescence intensities at 520 nm in the presence and absence of T1, respectively. Error bars show the standard deviations of three independent experiments.

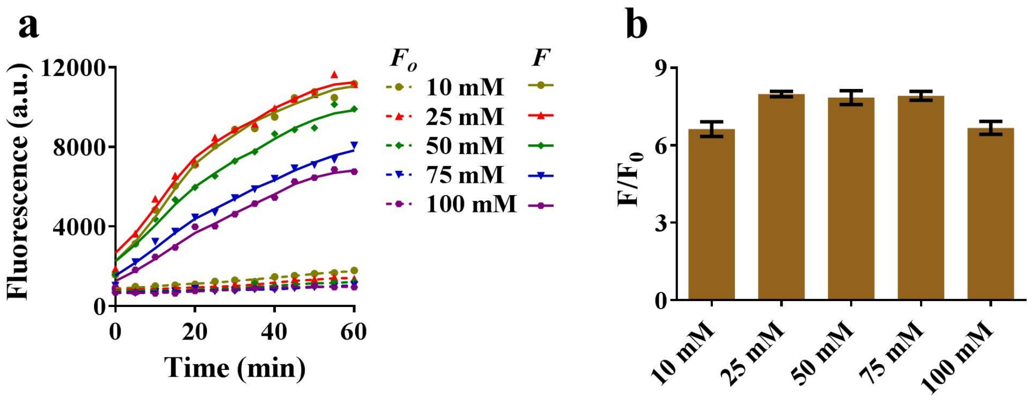

Fig. S6 Effect of Mg^{2+} concentration on the operation of the nanomachine. (a) Real-time fluorescence intensity was measured for 100 μL reaction system containing 0.1 nM nanomachine (S2/R1/A6-AuNP), 5 mM ATP and Mg^{2+} at different concentrations (10 mM, 25 mM, 50 mM, 75 mM and 100 mM). The solid and dashed lines represent fluorescence intensity in the presence and absence of 5 nM T1, respectively. (b) Fluorescence ratios (F/F_0) derived from the data (A), where F and F_0 are the fluorescence intensities at 520 nm in the presence and absence of T1, respectively. Error bars show the standard deviations of three independent experiments.

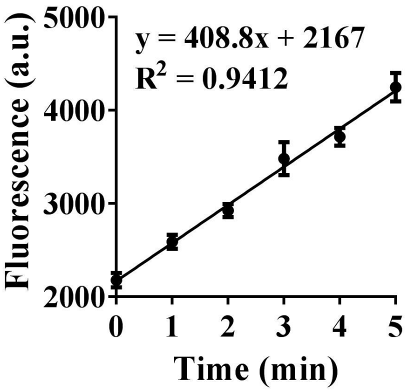

Fig. S7 Caculation of the initial rate of the proposed nanomachine by monitoring the fluorescence increases every 1 min for the first 5 min. Error bars show the standard deviations of three independent experiments.

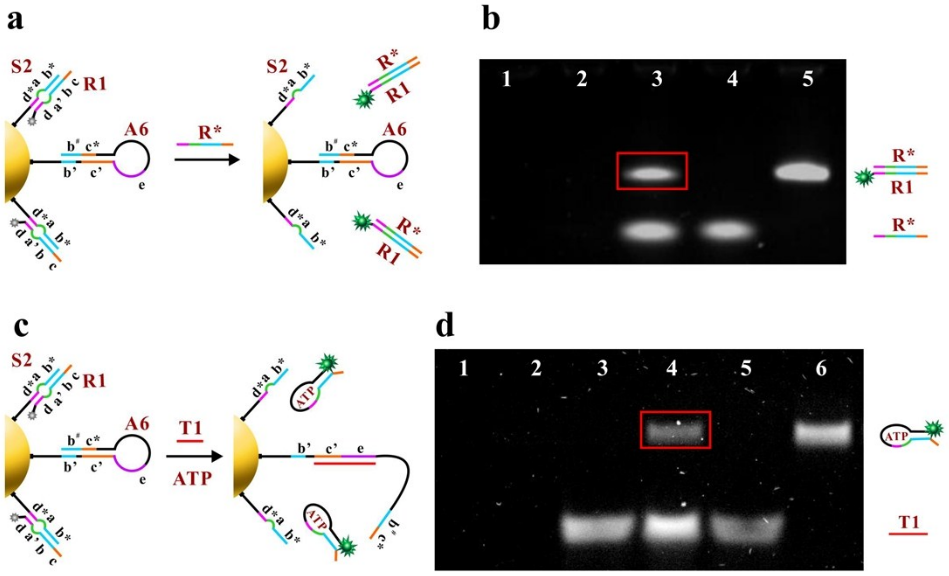

Fig. S8 (a) The schematic illustration of the reaction of the proposed nanomachine in the presence of R*. (b) Non-denaturing PAGE to analyze the collected supernatants of reaction mixtures with different components. Lane 1: the collected supernatant of S2/R1/A6-AuNP, lane 2: the collected supernatant of the mixture of S2/R1/A6-AuNP and ATP, lane 3: the collected supernatant of the mixture of S2/R1/A6-AuNP and R*, lane 4: R*, lane 5: mixture of R* and R1. (c) The schematic illustration of the motion of the proposed nanomachine in the presence of ATP and T1. (d) Non-denaturing PAGE to analyze the supernatants of reaction mixtures with different components. Lane 1: the collected supernatant of S2/R1/A6-AuNP, lane 2: the collected supernatant of the mixture of S2/R1/A6-AuNP and ATP, lane 3: the collected supernatant of the mixture of S2/R1/A6-AuNP and T1, lane 4: the collected supernatant of mixture of S2/R1/A6-AuNP, ATP and T1, lane 5: T1, lane 6: mixture of ATP and R1.

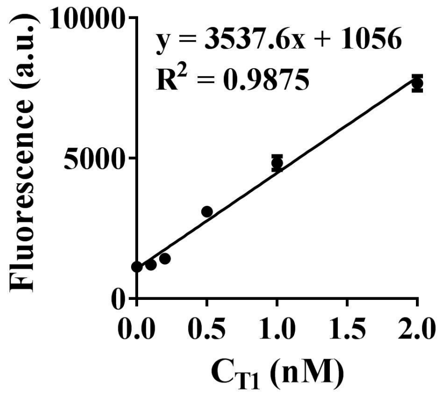

Fig. S9 The regression curve shows the fluorescence intensity as a function of T1 concentration. Error bars show the standard deviations of three independent experiments.

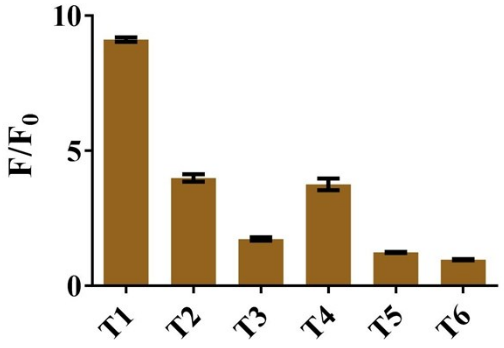

Fig. S10 Fluorescence ratios (F/F_0) derived from the data shown in Fig. 3b, where F and F_0 are the fluorescence intensities at 520 nm in the presence and absence of target, respectively. Error bars show the standard deviations of three independent experiments.

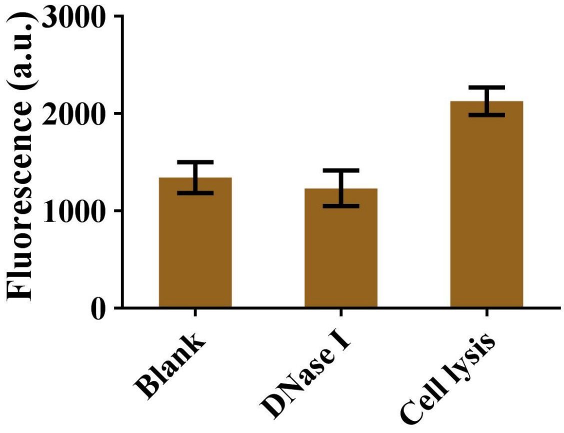

Fig. S11 Stability investigation of our nanomachine in DNase I and cell lysis. Blank represents 0.1 nM S2/R1/A6-AuNP in 1×PBS. DNase I represents 0.1 nM S2/R1/A6-AuNP in 1×PBS containing 50 U/L DNase I. Cell lysis represents 0.1 nM S2/R1/A6-AuNP in 1×PBS containing 50% (volume ratio) HeLa cell lysis.

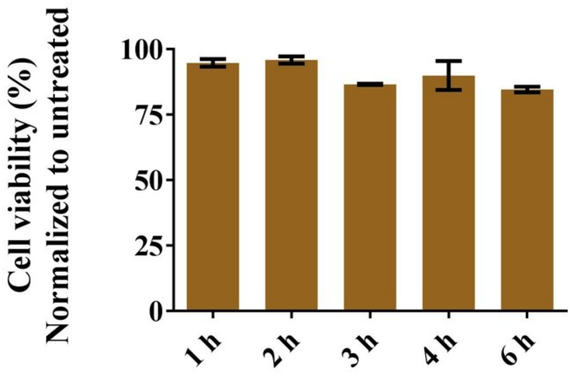

Fig. S12 Cytotoxicity analysis of the uptake of 0.2 nM nanomachine (S2/R1/A6-AuNP) in HeLa cells by CCK-8 assay. Error bars show the standard deviations of three independent experiments.

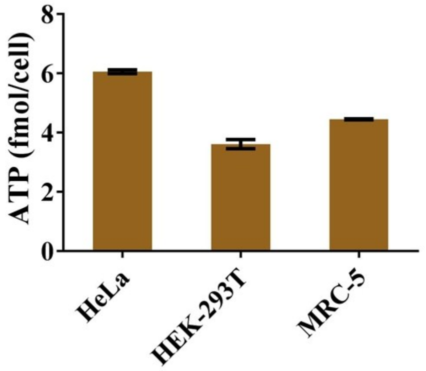

Fig. S13 The molar quantities of ATP in a single cell using ATP bioluminescent assay kit.

Table S2. Comparison of the needed incubation time for our method and other methods for the miRNA imaging

Method	Detection target and cell lines	Incubation time	Reference
Multifuctional SnO_2 nanoprobe	miR-21 (HeLa)	3 h	4
Gold@polydopamine core-shell nanoprobe	miR-29b and miR-31 (hMSCs)	24 h	5
Gold nanoparticle based hairpin-locked-DNAzyme probe	miR-141 (LOVE-1, SMMC-7721, 22Rv1, and HeLa)	8 h	6
Binary system based on nanobeacons	miR-21 (MCF-7)	90 min	7
Cascade hybridization reaction	miR-21 (HeLa, HEK-293,	4 h	8

	and MRC-5)		
DNA programmed nanoparticle disassembly	miR-21 (HeLa, MCF-7, MDA-MB-231, and HEK-293 cells)	6 h	9
Genetically encoded fluorescent RNA sensor	miR-21 (MCF-7, HeLa, and L02)	24 h	10
Nano metal–organic framework	miR-21 (MCF-7 and MDA-MB-231)	14 h	11
Entropy-driven DNA nanomachine	miR-21 (MCF-7, HeLa, HEK-293, and MRC-5)	5 h	1
MicroRNA-initiated DNAzyme motor	miR-10b (MDA-MB-231, MCF-10b, and MCF-7)	2 h incubation with motor and 1 h incubation with Mn^{2+}	12
ATP-powered DNA nanomachine	miR-21 (HeLa, HEK-293T, and MRC-5)	80 min	This work

References

1 C. P. Liang, P. Q. Ma, H. Liu, X. G. Guo, B. Yin and B. C. Ye, *Angew. Chem. Int. Ed.* 2017, **56**, 9077.

2 A. Mateus, P. Matsson and P. Artursson, *Mol. Pharmaceut.* 2013, **10**, 2467.

3 W. Zhou, Y. F. Tian, B. C. Yin and B. C. Ye, *Anal. Chem.* 2017, **89**, 6120.

4 H. Dong, J. Lei, H. Ju, F. Zhi, H. Wang, W. Guo, Z. Zhu and F. Yan, *Angew. Chem. Int. Ed.* 2012, **51**, 4607.

5 C. K. Choi, J. Li, K. Wei, Y. J. Xu, L. W. Ho, M. Zhu, K. K. To, C. H. Choi and L. Bian, *J. Am. Chem. Soc.* 2015, **137**, 7337.

6 Y. Yang, J. Huang, X. Yang, X. He, K. Quan, N. Xie, M. Ou and K. Wang, *Anal. Chem.* 2017, **89**, 5850.

7 R. C. Qian, Y. Cao and Y. T. Long, *Anal. Chem.* 2016, **88**, 8640.

8 Z. Cheglakov, T. M. Cronin, C. He and Y. Weizmann, *J. Am. Chem. Soc.* 2015, **137**, 6116.

9 X. He, T. Zeng, Z. Li, G. Wang and N. Ma, *Angew. Chem. Int. Ed.* 2016, **55**, 3073.

10 Z. M. Ying, Z. Wu, B. Tu, W. Tan and J. H. Jiang, *J. Am. Chem. Soc.* 2017, **139**, 9779.

11 Y. Wu, J. Han, P. Xue, R. Xu and Y. Kang, *Nanoscale* 2015, **7**, 1753.

12 H. Peng, X. F. Li, H. Zhang and X. C. Le, *Nat. Commun.* 2017, **8**, 14378.
